# Adiposity measured by body roundness index is significantly associated with increased stroke prevalence: a population-based cross-sectional study

**DOI:** 10.3389/fendo.2026.1761630

**Published:** 2026-04-01

**Authors:** Shengming Huang, Zhiwei Huang, Jin Wang, Jirui Cai, Li Guo, Jianwei Xiong, Guirang Zhao, Qiaotao Xie, Yijun Song, Haoran Wang

**Affiliations:** 1 Tianjin Medical University General Hospital, Tianjin, China; 2 State Key Laboratory of Experimental Hematology, National Clinical Research Center for Blood Diseases, Haihe Laboratory of Cell Ecosystem, Institute of Hematology & Blood Diseases Hospital, Chinese Academy of Medical Sciences & Peking Union Medical College, Tianjin, China; 3 Luohe Central Hospital, Luohe Medical College, Luohe, China; 4 Henan University College of Medicine, Kaifeng, China; 5 Luohe Center for Disease Control and Prevention, Luohe, China; 6 Haihe Laboratory of Cell Ecosystem, Tianjin Medical University General Hospital, Tianjin, China

**Keywords:** anthropometry, body roundness index, cross-sectional study, population study, screening triage, stroke prevalence

## Abstract

**Background:**

Stroke remains a major cause of disability and death. While central adiposity may contribute to vascular risk, the role of the body roundness index (BRI) (a waist-height-derived anthropometric indicator) in community screening populations is not well defined. We therefore investigated the association between BRI and prevalent stroke in a community-based screening sample.

**Methods:**

Using data from a ChinaHEART cohort branch (6,858 adults), BRI was calculated from anthropometric measurements. Prevalent stroke was ascertained by self-reported physician diagnosis. Receiver operating characteristic (ROC) analysis was used to determine the optimal BRI cutoff. Logistic regression, adjusted for age, sex, marital status, smoking, alcohol use, hypertension, diabetes, blood pressure, fasting glucose, and lipid parameters, was performed to examine the association between BRI and prevalent stroke. Restricted cubic spline (RCS) analysis and subgroup/interaction analyses were further conducted.

**Results:**

Among 6,858 participants, 192 (2.8%) reported prior stroke. The bootstrap-derived BRI cutoff was 4.597 (95% CI 4.149–4.798). High BRI (≥4.6) was associated with higher odds of prevalent stroke in the fully adjusted model (OR 1.766, 95% CI 1.279–2.438). BRI alone showed limited discrimination (AUC 0.584), while multivariable models achieved higher AUC (0.740 in the fully adjusted model). Adding BRI produced ΔAUC increases. RCS showed no evidence of nonlinearity.

**Conclusions:**

Our findings support BRI as a simple, non-invasive anthropometric measure that may offer complementary discriminatory information for community screening/triage; prospective studies with validated outcomes are warranted to confirm clinical utility.

## Introduction

Stroke remains a leading cause of disability and mortality worldwide (1, 2), with a significant burden in China due to its rapidly aging population and rising prevalence of modifiable risk factors such as hypertension, diabetes, and obesity (3, 4). Beyond traditional vascular risk factors, contemporary stroke research has highlighted the etiologic and pathobiological heterogeneity of the disease, accompanied by rapid advances in mechanistic understanding (5–11). Traditional determinants including age, gender, blood pressure, and lipid profiles are well-established in stroke risk profiling and prevalence assessment (12), however, they may not fully capture the contribution of body composition-particularly fat distribution-to cerebrovascular disease (13, 14). Accordingly, anthropometric indices have gained increasing attention as simple and cost-effective tools to characterize body shape and adiposity patterns in cardiovascular research (15, 16).

Body mass index (BMI) is widely used to quantify overall adiposity (16), yet it does not distinguish fat distribution and may underestimate the impact of central adiposity on stroke-related pathways. Central/visceral adiposity is metabolically active and is linked to chronic inflammation, insulin resistance, endothelial dysfunction, and atherosclerotic/thrombotic processes that are mechanistically relevant to stroke (13–15). Thus, waist–height–based indices have been proposed to better reflect body roundness and central adiposity relative to BMI alone (17, 18). The Body Roundness Index (BRI), derived from waist circumference (WC) and height, was developed from an original geometrical model to approximate body roundness and has been validated against body fat and visceral adiposity proxies (17). Compared with conventional measures, BRI may better capture central adiposity and related metabolic risk (17, 19).

Although prior population studies have linked BRI to stroke-related outcomes, evidence from large community-based cardiovascular screening programs with standardized anthropometry and rich cardiometabolic profiling remains limited. In this ChinaHEART Luohe screening sample, we (i) estimated a bootstrap-derived operational BRI cutoff for prevalent stroke classification, (ii) quantified the incremental change in discrimination when adding BRI to age/sex and fully adjusted models (ΔAUC), and (iii) explored potential effect modification by blood pressure and metabolic factors. These analyses aim to clarify the pragmatic role of BRI as an accessible anthropometric correlate of prevalent stroke in a real-world screening context (20–22).

## Methods

### Study design and population

We performed a cross-sectional analysis using data from the Luohe site of the ChinaHEART project (23, 24), an ongoing nationwide program for cardiovascular risk screening and management. Between November 2, 2021 and February 20, 2022, residents of Luohe City (central China) were invited to undergo standardized cardiovascular health assessments. Eligible participants were 35–75 years old at the time of screening (birth years 1946-1986), had lived in the catchment area for at least 6 months, and provided written informed consent. Individuals were excluded if key questionnaire items or clinical measurements were missing, if questionnaire responses were internally inconsistent (e.g., “unclear” or conflicting answers), or if the fasting period before blood sampling was less than 8 hours. Prevalent stroke was defined as self-reported physician-diagnosed stroke; participants with a prior physician diagnosis were included and classified as prevalent cases regardless of current symptoms. After these exclusions, 6,858 participants with complete data on BRI and stroke history were included in the present analysis. The current report followed the Strengthening the Reporting of Observational Studies in Epidemiology (STROBE) statement (25).

### Data collection and measurements

Trained healthcare staff collected data using a unified protocol derived from the ChinaHEART program. At the screening visit, participants completed interviewer-administered questionnaires and underwent physical examinations and fasting blood tests. The questionnaires includes.

sociodemographic information: age, sex, marital status, education and occupation; Lifestyle factors: current smoking (yes/no), alcohol consumption (yes/no); Medical history: self-reported physician-diagnosed stroke, hypertension, diabetes, dyslipidemia, and coronary heart disease; Medication use: current use of antihypertensive drugs, glucose-lowering agents, lipid-lowering drugs, and antiplatelet agents. Anthropometric measurements were performed with participants wearing light clothing and no shoes. Height was measured to the nearest 0.1 cm using a wall-mounted stadiometer, with participants standing upright, feet together, arms relaxed at the sides, and head positioned in the Frankfort horizontal plane. Weight was recorded to the nearest 0.1 kg using a calibrated scale. Body mass index (BMI) was calculated as weight (kg) divided by height squared (m²). Waist circumference (WC) was measured with a non-stretchable tape at the level of the umbilicus, with the tape held horizontally and in light contact with the skin, avoiding compression of soft tissue. Each measurement was taken at the end of a normal expiration, and recorded to the nearest 0.1 cm. Blood pressure was measured on the right arm with an automated oscillometric device after the participant had been seated quietly for at least 5 minutes, with back supported and feet flat on the floor. Two measurements were taken at 1-minute intervals; if the systolic readings differed by more than 10 mmHg, a third measurement was obtained. The mean of the last two readings was used in the analysis for both systolic and diastolic blood pressure. After an overnight fast of at least 8 hours, venous blood samples were collected. Fasting plasma glucose and serum lipids—including total cholesterol (TC), high-density lipoprotein cholesterol (HDL-C), low-density lipoprotein cholesterol (LDL-C), and triglycerides (TG)-were measured in the local hospital laboratory using standard enzymatic methods. All assays were performed on routinely calibrated analyzers in accordance with the manufacturer’s instructions.

### Body roundness index and other variables

Body Roundness Index (BRI) was used as the main exposure variable. BRI was calculated from WC and height using the original geometrical formula: BRI = 364.2 − 365.5 × √[1 − (WC/(2 × π × height/2))²], where WC and height are expressed in meters. Higher BRI values indicate a rounder body shape and greater central adiposity. The primary outcome was prevalent stroke, defined as a self-reported history of stroke diagnosed by a physician. Based on interview data, participants were categorized as having or not having a prior stroke. Secondary variables included traditional cardiovascular risk factors: age, sex, smoking status, alcohol use, history of hypertension and diabetes, systolic and diastolic blood pressure, fasting glucose, and lipid profile (TC, HDL-C, LDL-C, TG).

### Statistical analysis

Continuous variables are presented as medians with interquartile ranges (IQRs), and categorical variables as counts with percentages. Baseline characteristics were compared between BRI categories using the Wilcoxon rank-sum test for continuous variables and the chi-square test for categorical variables. To derive a clinically useful threshold for BRI in relation to stroke, we used receiver operating characteristic (ROC) analysis with 1,000-bootstrap resampling. For each bootstrap sample, the Youden index (sensitivity + specificity − 1) was computed to identify the BRI value that best separated participants with and without stroke. The final cutoff was defined as the median of these bootstrap-derived thresholds, with a bias-corrected 95% confidence interval. Participants were then classified into a low-BRI group and a high-BRI group according to this cutoff. We first evaluated the discriminatory ability of BRI for identifying prevalent stroke using ROC curves and areas under the curve (AUCs). Three nested logistic regression models were constructed: Model 1: BRI as the only predictor; Model 2: BRI plus age and sex; Model 3: fully adjusted model including BRI, age, sex, marital status, current smoking, alcohol use, history of hypertension and diabetes, systolic and diastolic blood pressure, fasting glucose, TC, HDL-C, LDL-C, and TG. For BRI, we examined both its continuous form (per unit increase) and the binary classification based on the optimal cutoff. Odds ratios (ORs) and 95% confidence intervals (CIs) were estimated for the association between BRI and stroke in each model. To explore the shape of the association between BRI and stroke, we fitted logistic regression models with BRI modeled using restricted cubic spline (RCS) with 3 knots. We assessed departure from linearity by comparing models with and without spline terms using likelihood ratio tests. Pre-specified subgroup analyses were conducted to evaluate whether the BRI-stroke association differed across strata of sex (male vs. female), age (<60 vs. ≥60 years), current smoking (yes vs. no), alcohol use (yes vs. no), hypertension (yes vs. no), and diabetes (yes vs. no). Interaction terms between BRI and each stratifying variable were introduced into the fully adjusted model, and p values for interaction were obtained from likelihood ratio tests. We also examined potential effect modification by blood pressure and lipid parameters. Continuous interaction terms between BRI and systolic blood pressure, diastolic blood pressure, TC, HDL-C, LDL-C, and TG were added separately to the full model, and their significance was tested. For visualization, predicted ORs of stroke across the BRI range were plotted at different levels of blood pressure and lipids. All analyses were performed using R (version 4.4.2; R Foundation for Statistical Computing, Vienna, Austria). A two-sided p value <0.05 was considered statistically significant.

To address the incremental discriminative contribution of BRI beyond established markers, we additionally compared Model 2 and Model 3 with versus without BRI using paired ROC analyses. The incremental change in discrimination was summarized as ΔAUC (AUC with BRI -AUC without BRI), and 95% confidence intervals for ΔAUC were obtained via 1,000 stratified bootstrap resamples. To address potential socioeconomic confounding, we performed a sensitivity incremental ROC analysis by additionally adjusting for socioeconomic indicators (education, occupation, and household income). Education was coded as a binary variable, while occupation and income were collapsed into parsimonious categories to reduce sparse cells.

## Results

**Figure 1:**
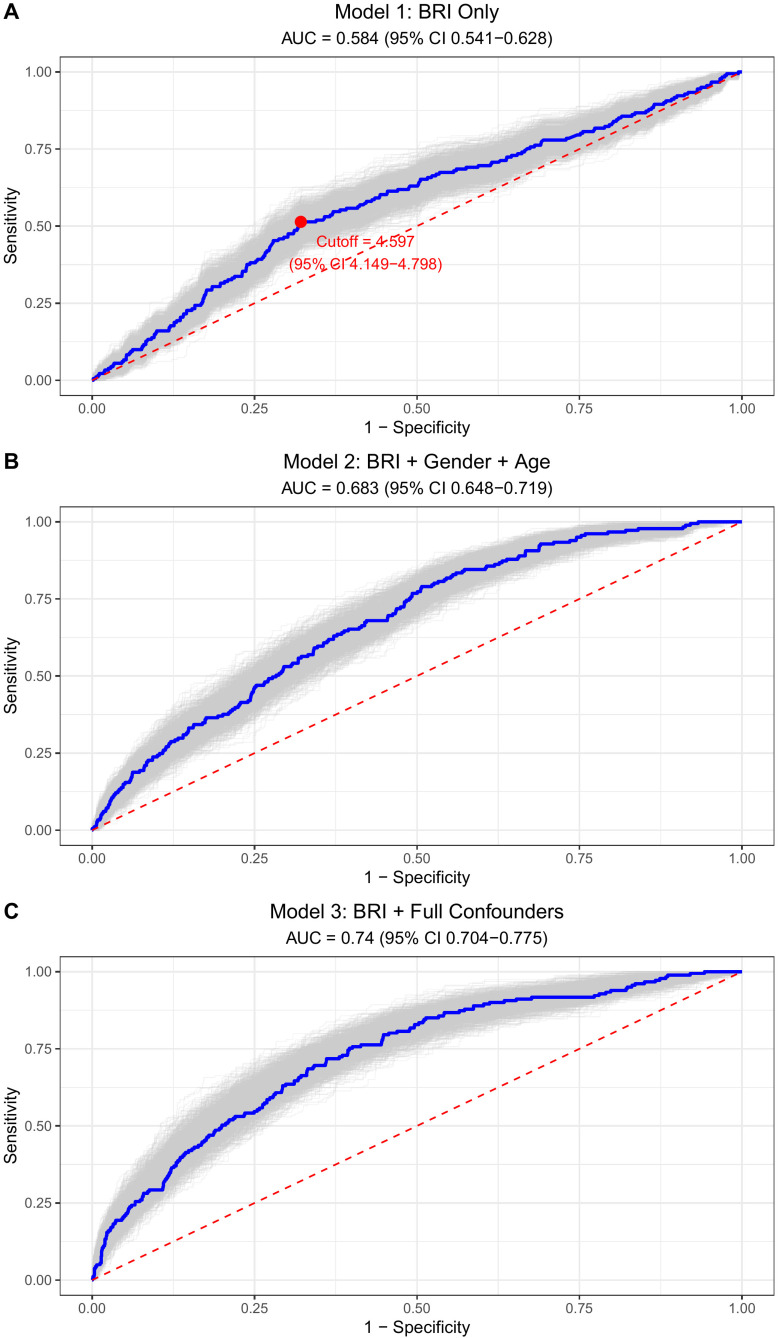
Receiver operating characteristic (ROC) curves for identifying prevalent stroke. **(A)** shows the model with BRI only; **(B)** shows the model with BRI, age, and gender; **(C)** shows the fully adjusted model.

A total of 6,858 participants were included in the study, of whom 192 had a history of stroke and 6,666 had no prior stroke history. ROC analysis (**Figure 1A**) identified a cutoff value of 4.597 (95% CI: 4.149-4.798), which effectively separated participants into low BRI (<4.6) and high BRI (≥4.6) groups. The baseline characteristics of the study population stratified by high and low BRI groups are presented in **Table 1**, with statistically significant differences observed in nearly all clinical features between the two groups. In absolute terms, prevalent stroke was observed in 96/2,261 (4.2%) participants in the high-BRI group compared with 96/4,597 (2.1%) in the low-BRI group (absolute difference, 2.1 percentage points). The discriminatory performance for prevalent stroke was evaluated using ROC analysis (**Figure 1**). BRI alone showed limited discrimination (Model 1 AUC = 0.584, 95% CI 0.541–0.628). When combined with age and sex (Model 2), discrimination increased (AUC = 0.683, 95% CI 0.648–0.719). In the fully adjusted model incorporating traditional cardiovascular risk factors (Model 3), the AUC was 0.740 (95% CI 0.704–0.775). To quantify the incremental discriminative value of adding BRI on top of established markers, we further compared models with versus without BRI (**Supplementary Figure 1**). In Model 2, adding BRI increased AUC from 0.670 (95% CI 0.633–0.704) to 0.683 (95% CI 0.648–0.719), corresponding to a ΔAUC of 0.012 (95% CI 0.001–0.035). In Model 3, adding BRI increased AUC from 0.735 (95% CI 0.697–0.769) to 0.740 (95% CI 0.704–0.775), corresponding to a ΔAUC of 0.004 (95% CI −0.000–0.017). To address potential socioeconomic confounding, we performed a sensitivity incremental ROC analysis by additionally adjusting Model 3 for education, occupation, and household income (**Supplementary Figure 1**). In this SES-adjusted model, adding BRI increased AUC from 0.765 (0.730−0.799) to 0.771 (0.736−0.806), corresponding to a ΔAUC of 0.005 (0.000−0.017). Overall, the incremental discrimination attributable to BRI remained modest after additional SES adjustment. These findings indicate that BRI alone has modest discriminatory ability, and its incremental contribution to discrimination is modest-particularly after comprehensive adjustment-despite the observed association between higher BRI and prevalent stroke. Taken together, these results suggest that BRI is associated with prevalent stroke and may provide additional discriminative information beyond conventional markers in this cross-sectional setting.

**Table 1:** Baseline characteristics by BRI status.

Variable	Overall (N = 6,858)	Low BRI (<4.6) (N = 4,597)	High BRI (≥4.6) (N = 2,261)	*P*-value
Baseline Information				
Age (years)	58 (51, 66)	57 (49, 66)	60 (53, 67)	<0.001
Gender (male)	2,586 (38%)	1,871 (41%)	715 (32%)	<0.001
Marital Status	6,087 (89%)	4,141 (90%)	1,946 (86%)	<0.001
Current Smoking	1,371 (20%)	979 (21%)	392 (17%)	<0.001
Alcohol Consumption	380 (5.5%)	228 (5.0%)	152 (6.7%)	0.003
History of Stroke	192 (2.8%)	96 (2.1%)	96 (4.2%)	<0.001
History of Diabetes	266 (3.9%)	121 (2.6%)	145 (6.4%)	<0.001
History of Hypertension	915 (13%)	418 (9.1%)	497 (22%)	<0.001
History of Dyslipidemia	286 (4.2%)	131 (2.8%)	155 (6.9%)	<0.001
Statin Use	207 (3.0%)	98 (2.1%)	109 (4.8%)	<0.001
Antiplatelet Medication Use	104 (1.5%)	49 (1.1%)	55 (2.4%)	<0.001
Diabetes Medication Use	387 (5.6%)	181 (3.9%)	206 (9.1%)	<0.001
Hypertension Medication Use	1,434 (21%)	677 (15%)	757 (33%)	<0.001
Lipid-Lowering Medication Use	462 (6.7%)	213 (4.6%)	249 (11%)	<0.001
Hypertension	3,615 (53%)	2,029 (44%)	1,586 (70%)	<0.001
Diabetes Mellitus	842 (12%)	401 (8.7%)	441 (20%)	<0.001
Coronary Heart Disease	110 (1.6%)	65 (1.4%)	45 (2.0%)	0.092
Body Size & Blood Pressure				
Height (cm)	159 (154, 166)	160 (155, 167)	157 (152, 163)	<0.001
Weight (kg)	64 (58, 72)	62 (55, 68)	70 (63, 77)	<0.001
Body Mass Index (kg/m²)	25.1 (23.0, 27.3)	23.8 (22.1, 25.5)	28.1 (26.4, 29.9)	<0.001
Waist Circumference (cm)	86 (80, 92)	82 (77, 86)	95 (91, 100)	<0.001
Systolic Blood Pressure (mmHg)	137 (126, 150)	134 (124, 148)	145 (132, 152)	<0.001
Diastolic Blood Pressure (mmHg)	84 (77, 91)	82 (76, 89)	87 (79, 94)	<0.001
Heart Rate (bpm)	76 (70, 83)	75 (70, 82)	76 (70, 83)	0.016
Biochemical Information				
Total Cholesterol (mmol/L)	4.82 (4.12, 5.50)	4.78 (4.09, 5.41)	4.88 (4.20, 5.67)	<0.001
HDL Cholesterol (mmol/L)	1.43 (1.23, 1.66)	1.45 (1.25, 1.69)	1.37 (1.17, 1.59)	<0.001
Triglycerides (mmol/L)	1.51 (1.14, 2.03)	1.44 (1.09, 1.83)	1.75 (1.31, 2.41)	<0.001
LDL Cholesterol (mmol/L)	2.63 (2.07, 3.15)	2.65 (2.10, 3.10)	2.59 (2.01, 3.26)	0.808
Fasting Glucose (mmol/L)	5.40 (5.20, 6.00)	5.40 (5.10, 5.80)	5.70 (5.30, 6.40)	<0.001

**Figure 2:**
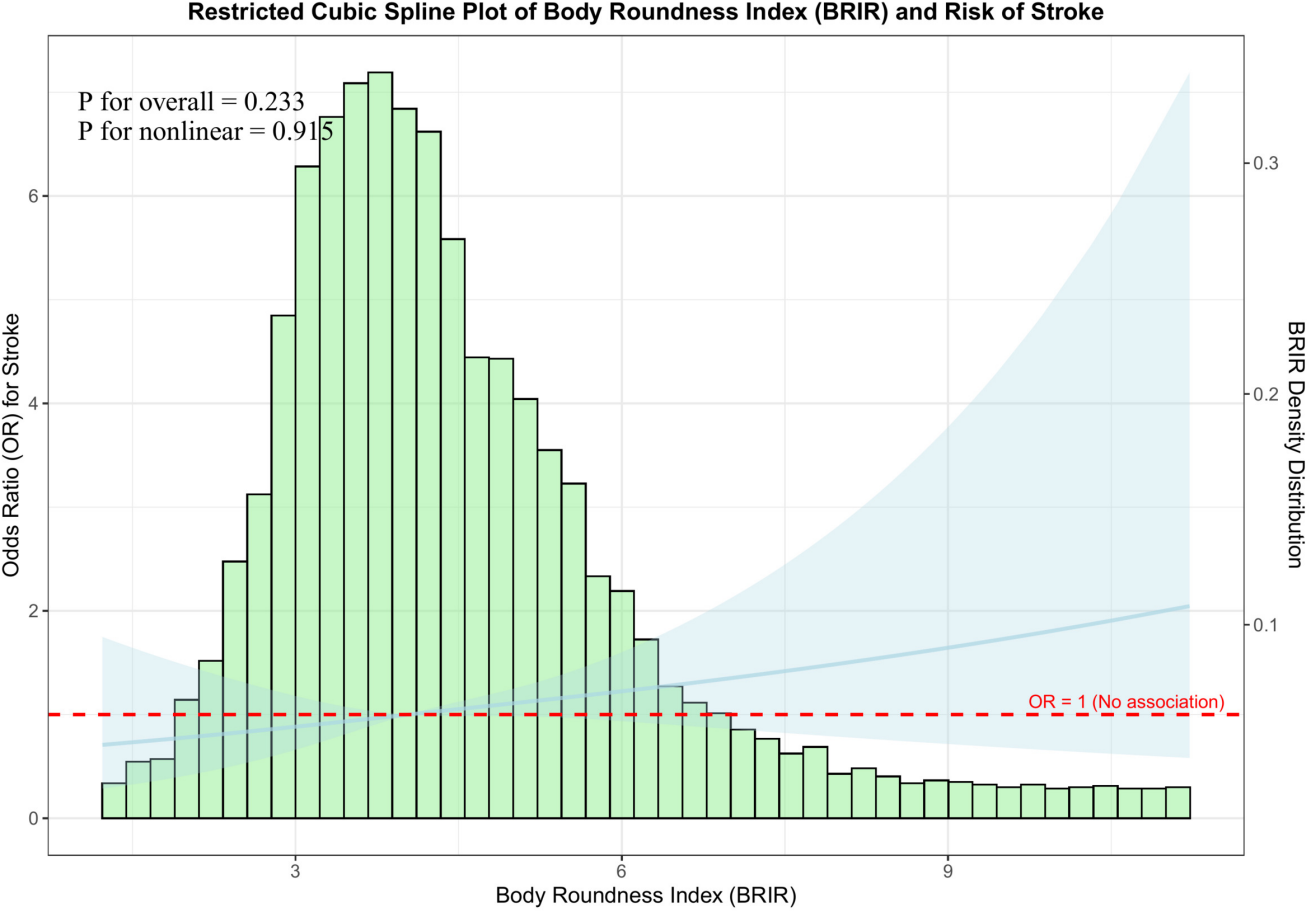
Dose-response relationship between Body Roundness Index (BRI) and stroke prevalence risk by restricted cubic spline (RCS).

To examine the nature of the relationship between BRI and stroke, RCS analysis was employed (**Figure 2**), which did not suggest evidence of nonlinearity in the BRI-stroke association (*P* for nonlinear = 0.915). Given attenuation in the fully adjusted model, the spline results should be interpreted as showing no clear departure from log-linearity across the observed range, with greater uncertainty at the extremes of BRI. Because observations were sparse at the upper tail of BRI (with wider confidence intervals), we cannot exclude a threshold or plateau pattern at very high BRI values despite the non-significant nonlinearity test. The overall association in the fully adjusted spline model was not statistically significant (*P* overall = 0.233), indicating attenuation after comprehensive adjustment.

**Table 2** shows logistic regression results for BRI-stroke associations across three models. For continuous BRI (per unit increase), Model 1 (crude) revealed a significant positive association (OR = 1.213, 95%CI: 1.093-1.347, *P* = 2.96×10^-4^). After adjusting for age and gender (Model 2), significance persisted (OR = 1.205, 95%CI:1.082-1.341, *P* = 7.05×10^-4^), but attenuated in the fully adjusted Model 3 (OR = 1.116, 95%: 0.989-1.259, *P* = 0.076). For categorical BRI (low BRI <4.6 as reference), high BRI (≥4.6) consistently correlated with higher stroke risk: Model 1 (OR = 2.079, 95%CI:1.560-2.771, *P* = 6.05×10^-7^), Model 2 (OR = 2.007, 95%CI:1.498-2.689, *P* = 3.02×10^-6^), and Model 3 (OR = 1.766, 95%CI:1.279-2.438, *P* = 5.50×10^-4^).

**Table 2:** Logistic regression assessing the association between BRI risk and stroke.

Variable	Model 1 (Crude)	Model 2 (adjusted for age and gender)	Model 3 (fully adjusted)
Continuous BRI (per unit increase)			
OR (95% CI)	1.213 (1.093-1.347)	1.205 (1.082-1.341)	1.116 (0.989-1.259)
*P*-value (Wald’s test)	2.96e-04	7.05e-04	0.076
Categorical BRI			
Low BRI (<4.6) [Reference]	Ref.	Ref.	Ref.
High BRI (≥4.6)			
OR (95% CI)	2.079 (1.560-2.771)	2.007 (1.498-2.689)	1.766 (1.279-2.438)
*P*-value (Wald’s test)	6.05e-07	3.02e-06	5.50e-04

Adjustment: age, sex, marital status, current smoking status, alcohol use, diabetes mellitus (DM), hypertension (HTN), systolic blood pressure (SBP), diastolic blood pressure (DBP), total cholesterol (TC), high-density lipoprotein cholesterol (HDL-C), triglycerides (TG), low-density lipoprotein cholesterol (LDL-C), and fasting glucose level.

**Figure 3:**
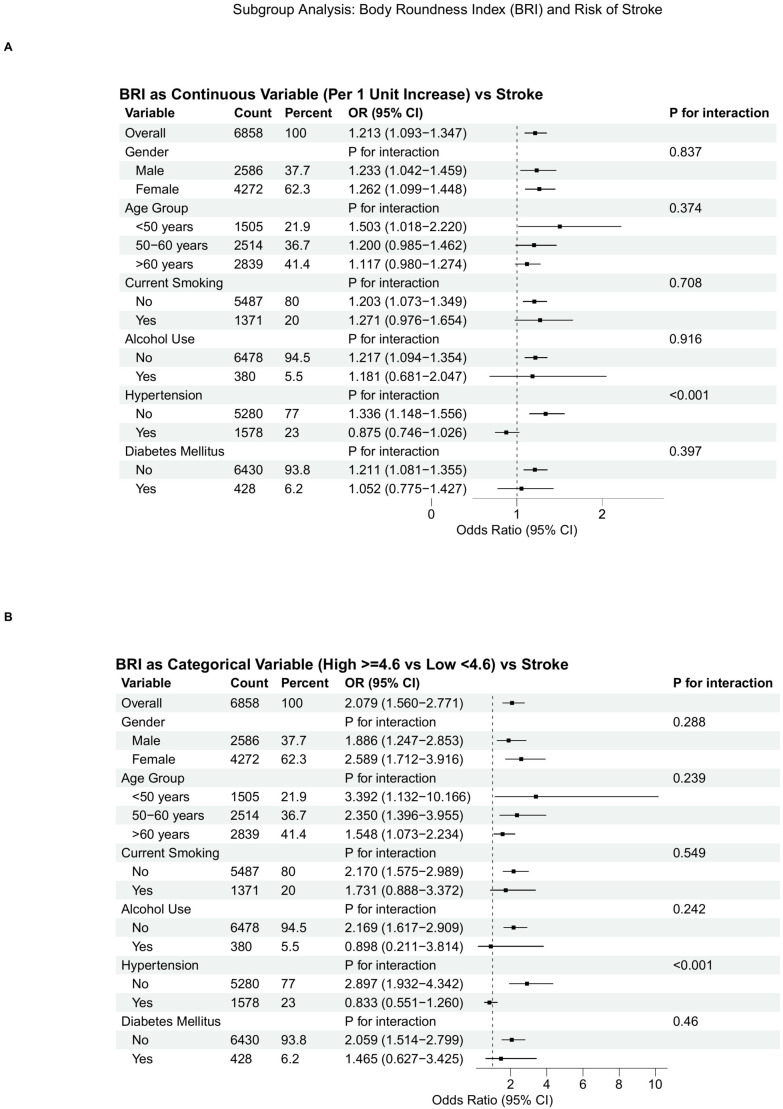
Subgroup analyses of the association between Body Roundness Index (BRI) and stroke prevalence. **(A)** shows the association with BRI as a continuous variable; **(B)** compares high vs. low BRI groups.

**Figure 4:**
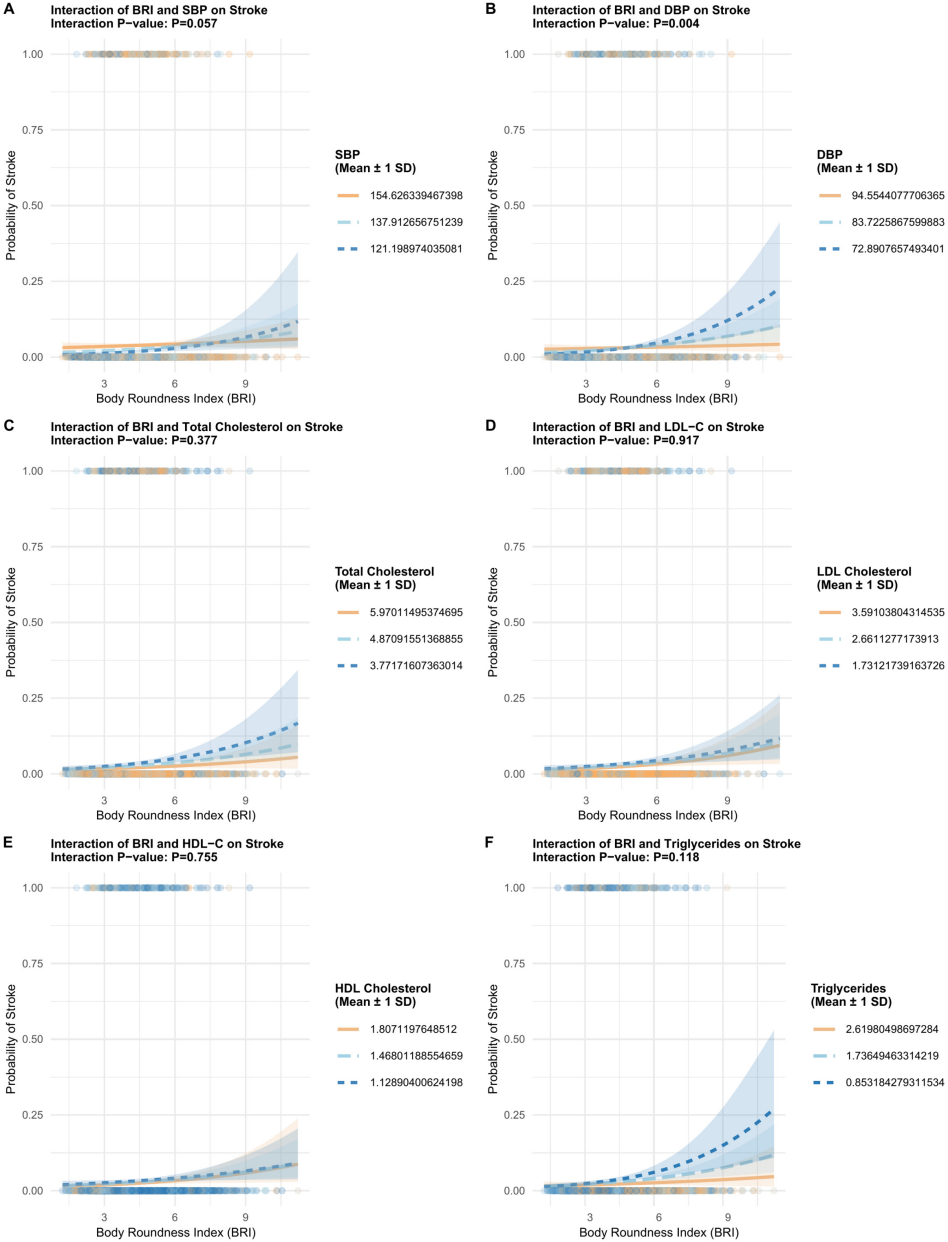
Interaction analyses of body roundness index (BRI) with blood pressure and lipid parameters in relation to prevalent stroke. **(A)** systolic blood pressure (SBP); **(B)** diastolic blood pressure (DBP); **(C)** total cholesterol (TC); **(D)** low-density lipoprotein cholesterol (LDL-C); **(E)** high-density lipoprotein cholesterol (HDL-C); and **(F)** triglycerides (TG). Each panel depicts the predicted probability of prevalent stroke across BRI values at three representative levels of the corresponding variable (mean ± 1 SD). Interaction P values are displayed in each panel.

Subgroup analysis (**Figure 3**) and interaction studies (**Figure 4**) shed further light on the heterogeneity of the BRI-stroke relationship. Analyses were conducted across key demographic and clinical variables, namely gender, age group, current smoking status, alcohol use, hypertension, and diabetes mellitus. The forest plot presented results of these subgroup analyses, investigating the BRI-stroke association using both continuous (**Figure 3A**) and categorical BRI models (**Figure 3B**) for interpretation. Notably, the interaction between BRI and hypertension history was statistically significant (*P* < 0.001). Detailed interaction plots (**Figure 4**) explored the interaction effects of blood pressure and lipid profiles on the BRI-stroke association. They revealed that diastolic blood pressure (DBP) played a significant modifying role (*P* = 0.004), while systolic blood pressure (SBP) exerted a borderline significant modifying effect (*P* = 0.057). Associations were generally directionally positive across most strata; however, effect estimates differed by hypertension status (*P* for interaction <0.001), suggesting potential effect modification. Interaction tests with lipid measures were not statistically significant; nonetheless, these tests may be underpowered and should be interpreted cautiously.

## Discussion

This study included 6,858 participants (192 with prevalent stroke history). ROC analysis identified a BRI cutoff of 4.597 (stratifying into <4.6 and ≥4.6), and showed limited discrimination for BRI alone (AUC = 0.584), whereas multivariable models achieved higher AUCs (Model 2 AUC = 0.683; Model 3 AUC = 0.740). Importantly, incremental ROC analyses (**Supplementary Figure 1**) indicated that adding BRI yielded an increase in discrimination beyond age/sex (ΔAUC=0.012) and additional gain beyond the fully adjusted model (ΔAUC=0.004). Fully adjusted logistic regression showed high BRI (≥4.6) significantly increased stroke prevalence (OR = 1.766). RCS analyses did not provide statistical evidence of nonlinearity (*P* for nonlinearity = 0.915); however, uncertainty increased at the upper tail of BRI, and a threshold/plateau pattern at very high BRI values cannot be ruled out and warrants confirmation in larger prospective samples with more extreme BRI values. The overall association in the spline model was not statistically significant (*P* for overall association = 0.233), consistent with attenuation in the fully adjusted model. Subgroup analyses revealed consistent BRI-stroke associations across most variables, except significant interaction with hypertension (*P* < 0.001); DBP significantly modified this association (*P* = 0.004), SBP borderline so (*P* = 0.057), but lipids did not, supporting an association between higher BRI and prevalent stroke, and a small incremental discrimination beyond established markers.

### Comparison with similar studies

Our study’s finding that a higher BRI is an anthropometric marker associated with prevalent stroke is broadly consistent with recent research investigating novel anthropometric indices in populations. A large-scale, cross-sectional study found that BRI was independently associated with stroke and may serve as an accessible anthropometric marker for identifying prevalent stroke (26).

A key aspect of our research is a broad monotonic pattern identified between BRI and stroke prevalence. This finding is consistent with a major prospective cohort study among Chinese adults, which not only linked elevated BRI to a higher risk of all-cause mortality but also specifically to cardiovascular disease (CVD) mortality (27). The consistent, graded increase in risk with rising BRI values observed in both our study and this large-scale mortality analysis suggests that BRI is not subject to the “obesity paradox” often seen with BMI. Our findings support BRI as a practical, non-invasive proxy of central adiposity that may complement conventional risk profiling, particularly when full risk-factor measurement is not feasible.

Furthermore, our results fit into the broader context of research highlighting the critical role of visceral fat in stroke pathogenesis. While BRI is an indirect measure, its strong association with stroke prevalence mirrors findings from studies using more direct, though complex, indices of visceral adiposity. For example, a study on Chinese patients with metabolic syndrome found that an elevated Chinese Visceral Adiposity Index (CVAI)-a metric specifically designed to estimate visceral fat-significantly increased the stroke prevalence (28). Our study complements this by offering BRI as a simpler, more accessible tool for clinical practice.

Finally, the emphasis on central obesity as a primary driver of risk is substantiated by a large-scale systematic review and meta-analysis of 72 prospective cohort studies. This extensive analysis demonstrated that central fatness, regardless of how it was measured (e.g., WC, WHR), is strongly and positively associated with all-cause and cause-specific mortality, including cardiovascular events (29). And this evidence further validates the clinical relevance of BRI as a anthropometric marker of central adiposity in stroke risk assessment.

### Clinical significance

From a discrimination perspective, adding BRI provided incremental improvement in model performance. In our bootstrap-based incremental ROC analyses, incorporation of BRI increased AUC when added to age and sex, and the direction of improvement was maintained after adjustment for conventional cardiovascular risk factors (**Supplementary Figure 1**). While the absolute change in AUC was modest-as expected in models that already include strong established predictors-these results support BRI as a simple anthropometric measure that can add complementary discriminatory information in community screening. Future prospective studies should evaluate clinical utility using complementary metrics (e.g., calibration, decision-curve analysis, and reclassification measures) and assess whether BRI improves screening triage in settings with limited access to biomarkers.

To further characterize vascular comorbidity in this screening population, we additionally reported baseline coronary heart disease (CHD) (**Table 1**). CHD prevalence was numerically higher in the high-BRI group, although the between-group difference was not statistically significant (*P* = 0.092). Given the cross-sectional design and questionnaire-based ascertainment, this finding should be interpreted descriptively; CHD may reflect shared atherosclerotic burden and risk-factor clustering rather than a factor that can be placed on a causal pathway in the present analysis. Future prospective studies with adjudicated outcomes are needed to clarify temporal relationships among central adiposity, CHD, and stroke.

To aid clinical interpretation of effect size, the observed prevalence of stroke was approximately two-fold higher in the high-BRI group than in the low-BRI group (4.2% vs 2.1%). In the fully adjusted model, high BRI remained associated with higher odds of prevalent stroke (OR 1.766, 95% CI 1.279–2.438), which should be interpreted as an association in a cross-sectional setting rather than a measure of future risk or treatment effect. The clinical implications of this study are particularly significant in the context of stroke prevention and vascular risk management (30). The association remained after multivariable adjustment (particularly for the dichotomized BRI), suggesting BRI may serve as a simple adjunct for preliminary screening triage, rather than materially improving discrimination in fully adjusted assessment models. Given its ease of measurement (calculated using anthropometric and physiological data), BRI may be a practical alternative or complement to more complex or expensive tests, such as MRI-based visceral fat assessment or advanced lipid profiling (31, 32). This is especially important in resource-limited settings, where high-cost and highly specialized diagnostic tools may not be widely accessible. Furthermore, the dichotomization of the population into high and low BRI groups using the optimal cutoff of 4.6 facilitates a pragmatic stratification for screening/triage prioritization (e.g., identifying individuals who may benefit from comprehensive vascular risk assessment and counseling), pending prospective validation (32).

The interaction between BRI and hypertension/blood pressure is particularly noteworthy, as it suggests that the BRI-stroke association may be modified by blood pressure metrics. This moderator effect has important implications for hypertension management, emphasizing the need to consider body composition alongside traditional vascular risk factors. The consistent trend of the association between BRI and stroke prevalence across the entire population range suggesting that BRI is a broadly applicable marker. These findings support that BRI could be considered as a simple adjunct anthropometric measure in preliminary risk assessment; however, whether it meaningfully improves identification beyond comprehensive risk profiling requires prospective validation using complementary metrics (e.g., calibration, decision-curve analysis, and reclassification measures) (21, 22). In practice, BRI could be used as a low-cost triage signal during community screening: individuals with high BRI may be prioritized for comprehensive vascular risk assessment (blood pressure, glucose, lipids) and targeted counseling on modifiable risk factors. Given the observed effect modification by hypertension/blood pressure, integrating BRI into hypertension management workflows may help identify subgroups who merit closer follow-up, while prospective studies are required before recommending BRI-based treatment thresholds.

### Strengths and limitations

One of the major strengths of this study is the large and well-characterized study population derived from a branch of the ChinaHEART cohort, ensuring sufficient statistical power to explore the BRI-stroke association. All measurements were collected under a standardized protocol with trained staff and unified instruments, enhancing internal validity and reproducibility. The use of ROC analysis to determine the optimal cutoff value for BRI enhances methodological rigor, while RCS modeling enables a comprehensive evaluation of the nature of the association between BRI and stroke. The three logistic regression models, which incorporate increasing levels of adjustment (from crude to fully adjusted for traditional cardiovascular risk factors), further refine the association, minimize potential confounding, and improve the interpretability of results. Additionally, subgroup analysis provides critical insights into the interaction between BRI and key clinical/demographic variables (e.g., disease history, gender, age), offering a basis for tailoring stroke risk assessments to specific populations that may benefit from targeted interventions.

Despite these advantages, the study is not without limitations. First, as a cross-sectional design, the study may not establish a causal relationship between BRI and stroke, but rather suggest a correlation that requires further validation through longitudinal studies (33). While the statistical significance of the association is established, the direction of the relationship (whether BRI causes or co-occurs with stroke) remains uncertain and requires further investigation. Second, the use of self-reported data for lifestyle factors, such as smoking and alcohol consumption, may introduce a potential for reporting bias (34), which could affect the accuracy of the associations. Third, the focus on a single population from Luohe City may limit the applicability of the findings to other regions, ethnicities, or healthcare systems, the limited regional scope of the study prevents generalization to diverse populations, especially those with different socioeconomic backgrounds or health behaviors. Fourth, stroke history was based on self-reported physician diagnosis rather than adjudicated medical records or imaging. This may introduce recall bias and outcome misclassification (e.g., under-reporting or confusion with transient neurologic events), which could bias associations toward or away from the null. Similarly, key lifestyle covariates (smoking and alcohol use) were self-reported and subject to reporting bias. In addition, standardized information on atrial fibrillation (AF) was not collected in this screening dataset; therefore, we could not adjust for AF, an important stroke-related condition, and residual confounding by AF and related cardiac comorbidities may partly influence the observed associations.

## Conclusion

In this community-based screening sample, these findings support BRI as a simple, non-invasive anthropometric measure that may offer complementary discriminatory information for community screening, particularly where comprehensive biomarker profiling is not readily available. Prospective studies with adjudicated outcomes are warranted to evaluate clinical utility using complementary metrics beyond AUC.

## Data Availability

The raw data supporting the conclusions of this article will be made available by the authors, without undue reservation.

## Supplementary material

Supplementary Figure 1 Incremental ROC analyses comparing models with versus without BRI. **(A)** compares Model 2 (age + sex) with and without BRI. **(B)** compares the fully adjusted Model 3 and an SES-extended model (Model 3 + SES), each with versus without BRI. ΔAUC is reported for adding BRI within Model 3 and within Model 3 + SES. Grey curves represent 1,000 stratified bootstrap resamples. AUCs and ΔAUC (AUC with BRI-AUC without BRI) are reported with bootstrap 95% confidence intervals. In the SES extension, education was coded as a binary variable, occupation was collapsed into five groups (agriculture/manual; white-collar/professional; service/self-employed; not in labor force; other/unknown), and income was collapsed into five ordered categories (<10,000; 10,000–25,000; 25,001–50,000; 50,001–100,000; ≥100,001).

## References

[1] Feigin VL Brainin M Norrving B Martins S Sacco RL Hacke W . World stroke organization (WSO): global stroke fact sheet 2022. Int J Stroke. (2022) 17:18–29. doi: 10.1177/17474930211065917 34986727

[2] GBD 2019 Stroke Collaborators . Global, regional, and national burden of stroke and its risk factors, 1990-2019: a systematic analysis for the Global Burden of Disease Study 2019. Lancet Neurol. (2021) 20:795–820. doi: 10.1016/S1474-4422(21)00252-0 34487721 PMC8443449

[3] Tu WJ Zhao Z Yin P Cao L Zeng J Chen H . Estimated burden of stroke in China in 2020. JAMA Netw Open. (2023) 6:e231455. doi: 10.1001/jamanetworkopen.2023.1455 36862407 PMC9982699

[4] Wang W Jiang B Sun H Ru X Sun D Wang L . Prevalence, incidence, and mortality of stroke in China: results from a nationwide population-based survey of 480 687 adults. Circulation. (2017) 135:759–71. doi: 10.1161/CIRCULATIONAHA.116.025250 28052979

[5] Wang M Yan H Zhang Y Zhou Q Meng X Lin J . Accelerated biological aging increases the risk of short- and long-term stroke prognosis in patients with ischemic stroke or TIA. EBioMedicine. (2024) 111:105494. doi: 10.1016/j.ebiom.2024.105494 39662178 PMC11697706

[6] Liu W Yang M Wang N Liu X Wang C Shi K . Intracalvariosseous injection: an approach for central nervous system drug delivery through skull bone marrow with a preclinical research in stroke. EBioMedicine. (2025) 112:105568. doi: 10.1016/j.ebiom.2025.105568 39884187 PMC11830332

[7] Fang Y Zhu Z Zhang JH Shi FD Yang Q . The vascular-immune-neural network: a new pathophysiological paradigm and the dawn of cytoprotection in stroke. EBioMedicine. (2025) 118:105843. doi: 10.1016/j.ebiom.2025.105843 40618510 PMC12272477

[8] Schoels M Krumm L Nelde A Olma MC Nolte CH Scheitz JF . Artificial intelligence for prediction of atrial fibrillation in the stroke unit: a retrospective derivation validation cohort study. EBioMedicine. (2025) 118:105869. doi: 10.1016/j.ebiom.2025.105869 40752407 PMC12341230

[9] Zheng Y Tan X Wang X Mao R Guo J . Targeting ferroptosis with natural products in stroke: therapeutic mechanisms and translational opportunities. Front Pharmacol. (2025) 16:1586345. doi: 10.3389/fphar.2025.1586345 40510422 PMC12159007

[10] Sowmiya S Begum RF Dhivya LS Rajendran P Harikrishnan N Singh SA . Traditional, complementary, and integrative medicine in the management of ischemic stroke: a narrative review. Front Pharmacol. (2025) 16:1561688. doi: 10.3389/fphar.2025.1561688 40520194 PMC12163044

[11] Li W Wu J Hu Z Zhang J Ye G Luo F . The prospective approach for aptamers applied in the treatment and molecular diagnostics of ischemic stroke. Front Pharmacol. (2025) 16:1553337. doi: 10.3389/fphar.2025.1553337 40376266 PMC12079141

[12] O'Donnell MJ Xavier D Liu L Zhang H Chin SL Rao-Melacini P . Risk factors for ischemic and intracerebral hemorrhagic stroke in 22 countries (the INTERSTROKE study): a case-control study. Lancet. (2010) 376:112–23. doi: 10.1016/S0140-6736(10)60834-3 20561675

[13] Piché ME Tchernof A Després JP . Obesity phenotypes, diabetes, and cardiovascular diseases. Circ Res. (2020) 126:1477–500. doi: 10.1161/CIRCRESAHA.120.316101 32437302

[14] Neeland IJ Ross R Després JP Matsuzawa Y Yamashita S Shai I . Visceral and ectopic fat, atherosclerosis, and cardiometabolic disease: a position statement. Lancet Diabetes Endocrinol. (2019) 7:715–25. doi: 10.1016/S2213-8587(19)30084-1 31301983

[15] Ross R Neeland IJ Yamashita S Shai I Seidell J Magni P . Waist circumference as a vital sign in clinical practice: a Consensus Statement from the IAS and ICCR Working Group on Visceral Obesity. Nat Rev Endocrinol. (2020) 16:177–89. doi: 10.1038/s41574-019-0310-7 32020062 PMC7027970

[16] Powell-Wiley TM Poirier P Burke LE Després JP Gordon-Larsen P Lavie CJ . Obesity and cardiovascular disease: a scientific statement from the American Heart Association. Circulation. (2021) 143:e984–e1010. doi: 10.1161/CIR.0000000000000973 33882682 PMC8493650

[17] Thomas DM Bredlau C Bosy-Westphal A Mueller M Shen W Gallagher D . Relationships between body roundness with body fat and visceral adipose tissue emerging from a new geometrical model. Obes (Silver Spring). (2013) 21:2264–71. doi: 10.1002/oby.20408 23519954 PMC3692604

[18] Rico-Martín S Calderón-García JF Sánchez-Rey P Franco-Antonio C Martínez Alvarez M Sánchez Muñoz-Torrero JF . Effectiveness of body roundness index in predicting metabolic syndrome: a systematic review and meta-analysis. Obes Rev. (2020) 21:e13023. doi: 10.1111/obr.13023 32267621

[19] Liu Y Liu X Guan H Zhang S Zhu Q Fu X . Body roundness index is a superior obesity index in predicting diabetes risk among hypertensive patients: a prospective cohort study in China. Front Cardiovasc Med. (2021) 8:736073. doi: 10.3389/fcvm.2021.736073 34869638 PMC8638826

[20] Boehme AK Esenwa C Elkind MSV . Stroke risk factors, genetics, and prevention. Circ Res. (2017) 120:472–95. doi: 10.1161/CIRCRESAHA.116.308398 28154098 PMC5321635

[21] Damen JAAG Hooft L Schuit E Debray TPA Collins GS Tzoulaki I . Prediction models for cardiovascular disease risk in the general population: systematic review. BMJ. (2016) 353:i2416. doi: 10.1136/bmj.i2416 27184143 PMC4868251

[22] Pencina MJ Navar AM Wojdyla D Sanchez RJ Khan I Elassal J . Quantifying importance of major risk factors for coronary heart disease. Circulation. (2019) 139:1603–11. doi: 10.1161/CIRCULATIONAHA.117.031855 30586759 PMC6433489

[23] Lu J Xuan S Downing NS Wu C Li L Krumholz HM . Protocol for the China PEACE (Patient-centered evaluative assessment of cardiac events) million persons project pilot. BMJ Open. (2016) 6:e010200. doi: 10.1136/bmjopen-2015-010200 26729395 PMC4716208

[24] Wang R Yang Y Lu J Cui J Xu W Song L . Cohort profile: ChinaHEART (Health Evaluation And risk Reduction through nationwide Teamwork) Cohort. Int J Epidemiol. (2023) 52:e273–82. doi: 10.1093/ije/dyad074 37257881

[25] von Elm E Altman DG Egger M Pocock SJ Gøtzsche PC Vandenbroucke JP . The Strengthening the Reporting of Observational Studies in Epidemiology (STROBE) statement: guidelines for reporting observational studies. Lancet. (2007) 370:1453–7. doi: 10.1016/S0140-6736(07)61602-X 18064739

[26] Zhang P Sun X Jin H Zhang FL Guo ZN Yang Y . Comparison of the four anthropometric indexes and their association with stroke: a population-based cross-sectional study in Jilin Province, China. Front Neurol. (2019) 10:1304. doi: 10.3389/fneur.2019.01304 31920927 PMC6914861

[27] Lin H Jia X Yin Y Li M Zheng R Xu Y . Association of body roundness index with cardiovascular disease and all-cause mortality among Chinese adults. Diabetes Obes Metab. (2025) 27:2698–707. doi: 10.1111/dom.16272 39972403

[28] Liu Z Huang Q Deng B Wei M Feng X Yu F . Elevated Chinese visceral adiposity index increases the risk of stroke in Chinese patients with metabolic syndrome. Front Endocrinol (Lausanne). (2023) 14:1218905. doi: 10.3389/fendo.2023.1218905 37455909 PMC10339806

[29] Jayedi A Soltani S Zargar MS Khan TA Shab-Bidar S . Central fatness and risk of all-cause mortality: systematic review and dose-response meta-analysis of 72 prospective cohort studies. BMJ. (2020) 370:m3324. doi: 10.1136/bmj.m3324 32967840 PMC7509947

[30] Virani SS Alonso A Aparicio HJ Benjamin EJ Bittencourt MS Callaway CW . Heart disease and stroke statistics-2021 update: a report from the American Heart Association. Circulation. (2021) 143:e254–743. doi: 10.1161/CIR.0000000000000950 33501848 PMC13036842

[31] Albus C . Low socio-economic status: how can we prevent its negative impact on cardiovascular disease incidence and prognosis? Eur J Prev Cardiol. (2024) 31:38–9. doi: 10.1093/eurjpc/zwad308 37725917

[32] Arnett DK Blumenthal RS Albert MA Buroker AB Goldberger ZD Hahn EJ . 2019 ACC/AHA Guideline on the primary prevention of cardiovascular disease: a report of the American College of Cardiology/American Heart Association Task Force on Clinical Practice Guidelines. Circulation. (2019) 140:e596–646. doi: 10.1161/CIR.0000000000000678 30879355 PMC7734661

[33] Setia MS . Methodology series module 3: cross-sectional studies. Indian J Dermatol. (2016) 61:261–4. doi: 10.4103/0019-5154.182410 27293245 PMC4885177

[34] Althubaiti A . Information bias in health research: definition, pitfalls, and adjustment methods. J Multidiscip Healthc. (2016) 9:211–7. doi: 10.2147/JMDH.S104807 27217764 PMC4862344

