# Supplementary figures and images for: Adiposity measured by body roundness index is significantly associated with increased stroke prevalence: a population-based cross-sectional study
Source: Front Endocrinol (Lausanne). 2026 Apr 1;17:1761630. doi: 10.3389/fendo.2026.1761630 (PMC13081234; doi:10.3389/fendo.2026.1761630)

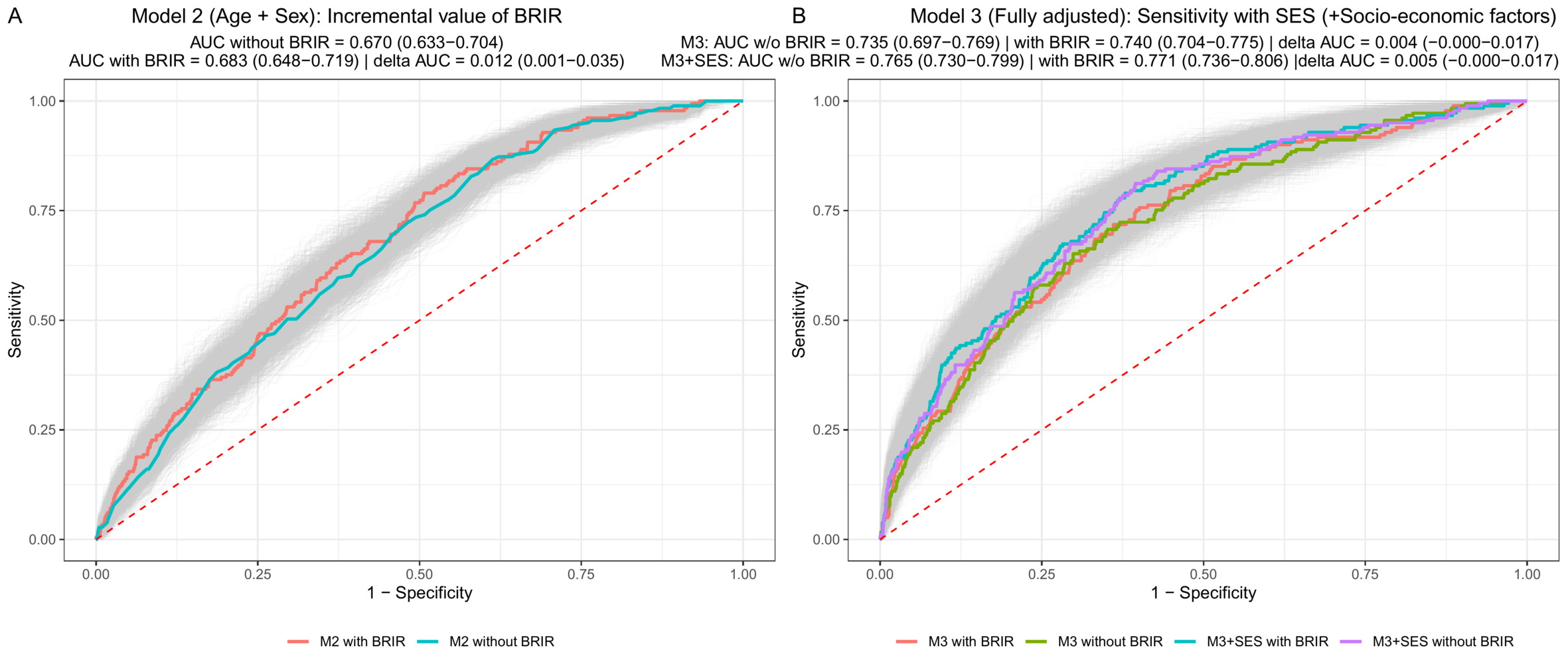

Supplement: Supplementary Figure 1 — Incremental ROC analyses comparing models with versus without BRI. (A) compares Model 2 (age + sex) with and without BRI. (B) compares the fully adjusted Model 3 and an SES-extended model (Model 3 + SES), each with versus without BRI. ΔAUC is reported for adding BRI within Model 3 and within Model 3 + SES. Grey curves represent 1,000 stratified bootstrap resamples. AUCs and ΔAUC (AUC with BRI-AUC without BRI) are reported with bootstrap 95% confidence intervals. In the SES extension, education was coded as a binary variable, occupation was collapsed into five groups (agriculture/manual; white-collar/professional; service/self-employed; not in labor force; other/unknown), and income was collapsed into five ordered categories (<10,000; 10,000–25,000; 25,001–50,000; 50,001–100,000; ≥100,001). [file Image1.jpeg]
